# Understanding the Mechanistic Connection between Dysbiosis and Polyamine Regulation in Oral and Intestinal Inflammation– Role of Tregs and Th17 Cells

**Published:** 2026

**Authors:** Pushpa Pandiyan

**Affiliations:** 1 Department of Biological Sciences, School of Dental Medicine, Case Western Reserve University, Cleveland, Ohio, 44106, USA; 2 Department of Pathology, Case Western Reserve University, Cleveland, Ohio, 44106, USA; 3 Department of Molecular Biology and Microbiology, School of Medicine, Case Western Reserve University, Cleveland, Ohio, 44106, USA; 4 Center for AIDS Research, Case Western Reserve University, Cleveland, Ohio, 44106, USA

**Keywords:** Inflammatory bowel disease, Intestinal inflammation, T cells

## Abstract

Periodontitis and inflammatory bowel disease (IBD) are chronic inflammatory conditions driven by dysbiotic microbial communities that subvert host mucosal immunity. Central to the immunopathology of both diseases is the disruption of the balance between regulatory T cells (Tregs) and T helper 17 (Th17) cells, an equilibrium that governs tissue homeostasis versus inflammatory destruction. Polyamines, principally putrescine (PUT), spermidine (SPD), and spermine (SPN), are emerging as critical immunometabolic regulators that modulate the plasticity and functional identity of both cell populations. Dysbiosis, particularly the enrichment of microbial communities that drive aberrant polyamine biosynthesis through ornithine decarboxylase (ODC)-dependent pathways, generates a polyamine-rich microenvironment that further dysregulates T cell function and promotes sustained inflammation. Experimental evidence demonstrates that mucosal polyamine levels correlate with dysbiosis, dysfunctional Treg (TregDys) expansion, and CD4^+^CD4^+^T cell hyperactivation, collectively contributing to and perpetuating chronic inflammation. The striking parallels between periodontitis and IBD reinforce the existence of conserved mechanisms linking mucosal dysbiosis to polyamine-driven immune dysregulation, with shared therapeutic implications for targeting the dysbiosis–T cell–polyamine axis to restore immune homeostasis. This review presents a unified mechanistic framework connecting oral and intestinal dysbiosis, polyamine dysregulation, and disruption of the Treg/Th17 balance, linking both diseases along the oral-gut axis.

## Introduction

Periodontitis and IBD are prevalent chronic inflammatory diseases arising at distinct mucosal sites-the oral cavity and the gastrointestinal tract, yet sharing fundamental pathogenic mechanisms rooted in dysbiosis-driven immune dysregulation. Periodontitis is characterized by progressive destruction of tooth-supporting structures and alveolar bone loss [1], while IBD [2], encompassing ulcerative colitis (UC) and Crohn's disease (CD), involves chronic mucosal inflammation and epithelial barrier disruption. In both conditions, the transition from a healthy, symbiotic microbial community to a dysbiotic, disease-driving one represents a pivotal pathogenic event that subverts host immunity and perpetuates tissue destruction [3-5]. At the immunological core of both diseases lies the dynamic balance between CD4^+^CD25^+^FOXP3^+^ Tregs and RORγt^+^IL-17A^+^ Th17 cells. The microbiome is a key determinant of Treg and Th17 cell heterogeneity and plasticity, defining the boundary between host defense and immunopathology. Dysbiosis disrupts this equilibrium by generating dysfunctional Tregs and subverting protective Th17 cells into inflammatory effectors, collectively perpetuating tissue destruction [6-13]. Polyamines are small polycationic aliphatic amines and are ubiquitous metabolites derived principally from the ornithine/arginine pathway via the rate-limiting enzyme ODC. Their positive charge at physiological pH enables electrostatic binding to negatively-charged nucleic acids, making them critical regulators of cell growth, proliferation, differentiation, gene regulation, and apoptosis [14,15]. These molecules are fundamental to cell proliferation, differentiation, RNA stability, and gene regulation, and their dual roles in pro- and anti-inflammatory immune responses are increasingly appreciated [16,17]. Critically, polyamines regulate T helper cell lineage fidelity through the eIF5A hypusination pathway, wherein SPD serves as the obligate substrate for post-translational modification of eukaryotic initiation factor 5A (eIF5A)-a modification essential for the translation of specific T cell effector transcripts [18,19]. The association between polyamines and periodontitis was established by Lamster *et al*. (1987) [20], who demonstrated higher levels of PUT concentrations in gingival crevicular fluid (GCF) than serum levels in untreated periodontitis, with concentrations declining substantially following treatment. Our laboratory was the first to demonstrate that polyamine dysregulation drives oral T cell dysfunction in HIV-infected individuals, establishing the PUT–eIF5A axis as a cardinal determinant of oral mucosal T helper dysregulation [7,21]. In IBD, dysregulation of polyamine metabolism is increasingly recognized as both a consequence of and contributor to chronic mucosal inflammation [22]. This review outlines the current mechanistic understanding of how dysbiosis and polyamine dysregulation converge to disrupt the Treg/Th17 axis in periodontitis and IBD, positioning polyamine-producing microbiota as a central mechanistic link between these two diseases along the oral-gut axis, and highlights the therapeutic implications of targeting this immunometabolic pathway to restore mucosal immune homeostasis.

## Tregs and Th17 Cells in Periodontitis and Inflammatory Bowel Disease

### Th17 cell biology: differentiation, function, and pathogenic plasticity

Th17 cells represent a distinct lineage of CD4^+^ T helper cells whose differentiation is governed by the master transcription factor ROR*γ*t, induced by a cytokine milieu comprising TGF-*β*, IL-6, IL-1*β*, IL-21, and IL-23 [23]. The canonical Th17 signature encompasses production of IL-17A, IL-17F, IL-21, IL-22, and GM-CSF—cytokines that collectively coordinate mucosal immunity, neutrophil mobilization, and epithelial barrier reinforcement [24]. Th17 cells serve dual functions in the oral mucosa. They contribute to host defense by promoting neutrophil recruitment, enhancing phagocytic activity, and maintaining mucosal immunity, but can also drive hyperinflammatory tissue damage. Their net impact depends on disease stage, cytokine environment, Treg function, and microbial context [10-13,21,25,26]. In the context of the dysbiotic oral microbiome, Th17 cells have emerged as central regulators of periodontal inflammation and bone remodeling [27]. Mechanistically, IL-17A signals on non-hematopoietic stromal targets—including gingival fibroblasts, epithelial cells, and endothelial cells—inducing NF-*κ*B and STAT-3 activation and production of IL-6, IL-8 (CXCL8), TNF-*α*, IL-*β*, IL-23, G-CSF, and chemokines CXCL1 and CXCL2. These mediators collectively sustain neutrophil recruitment, tissue destruction, and osteoclast activation [28,29]. Experimental therapies targeting Th17 cells have shown efficacy in murine periodontitis models; however, complete IL-17 inhibition may compromise host defense, emphasizing the need for context-specific immunomodulation rather than global Th17 suppression [27,30]. Fate-mapping studies have demonstrated that Th17 cells retain remarkable phenotypic plasticity, acquiring the capacity to produce IFN-*γ* in a manner strongly dependent on the degree of dysbiosis or infection [31,32]. The transcription factors T-bet and RUNX1 are required for the ontogeny of these pathogenic IFN-*γ*-producing Th17 cells. This heterogeneity distinguishes pathogenic Th17 cells from non-pathogenic and barrier-protective Th17 cells that co-express IL-10. In IBD, Th17 cells similarly play a dual role [33-37]. The IL-23/IL-17 axis drives intestinal inflammation in both UC and CD, with pathogenic Th17 cells contributing to mucosal barrier disruption, epithelial damage, and perpetuation of chronic inflammation [24]. The shared involvement of the IL-23/IL-17 axis across periodontitis and IBD underscores the conserved nature of Th17-driven immunopathology at mucosal surfaces [38].

### Regulatory T cells (Tregs): guardians of immune homeostasis

Tregs, defined by CD4^+^CD25^+^FOXP3^+^expression, are essential orchestrators of peripheral immune tolerance. Their suppressive mechanisms encompass deprivation of IL-2 from CD4^+^ effector cells, secretion of anti-inflammatory cytokines (IL-10, TGF-*β*, IL-35), contact-dependent inhibition via CTLA-4, and metabolic disruption of effector T cells [10-12,39-42]. Through these mechanisms, Tregs limit immunopathology and preserve mucosal tolerance. Clinical studies have reported accumulation of Tregs in moderate and advanced chronic periodontitis biopsies compared to gingivitis, with chemokines CCL17 and CCL22 recruiting Tregs to inflammatory sites via CCR4-dependent mechanisms [43,44]. However, a critical paradox has emerged: a small population of IL-17A^+^FOXP3^+^cells has been identified in periodontitis but not gingivitis, suggesting functional plasticity of Tregs transforming into inflammatory Th17-like cells within the periodontitis microenvironment [45,46]. However, the exact function of this transient population is unknown. Animal model studies reinforce the pathogenic consequences of Treg dysfunction: inhibition of Treg function by anti-GITR in *A. actinomycetemcomitans*-induced periodontitis resulted in alveolar bone resorption and increased inflammatory cell infiltration, accompanied by decreased IL-10, TGF-*β*, and CTLA-4. In experimental periodontitis models, downregulated FOXP3 expression and impaired Treg suppression of osteoclast differentiation further promoted Th17-driven bone loss, with hypermethylation of CpG sites in the FOXP3 locus identified as a potential epigenetic mechanism [47,48]. In the context of bone homeostasis, Tregs dose-dependently inhibit RANKL-dependent osteoclast formation through direct cell-cell contact via CTLA-4, with pit formation inhibited by up to 80% in co-culture systems. Although Treg-mediated expression of TGF-*β*, IL-4, and IL-10 contributed to but was not essential for this inhibitory effect, these findings position Tregs as critical brakes on bone-destructive processes in periodontitis [49]. In IBD, Tregs are major players in maintaining gut immune homeostasis [11,12,25,50-52]. However, Treg dysfunction contributes to disease progression. Impaired Treg suppressive capacity allows unchecked Th17 expansion, amplifying intestinal inflammation and mucosal injury [11,53]. The reciprocal relationship between Tregs and Th17 cells is governed by shared developmental signals: TGF-*β* promotes both FOXP3^+^Treg differentiation and, in combination with IL-6, ROR*γ*t^+^Th17 differentiation, positioning the cytokine environment as a critical determinant of the Treg/Th17 balance [10,11,13,34,35,54]. In the context of chronic viral infection, Tregs can also undergo dysbiosis-driven trans-differentiation, paradoxically expressing IFN-*γ* and losing suppressive function despite retaining FOXP3. This destabilized TregDys phenotype represents a cell caught between regulatory and effector identity, expressing high levels of the tissue remodeling marker amphiregulin (AREG) [55,56]. The precise contribution of dysbiosis to AREG^+^Treg destabilization in IBD remains an active area of investigation.

## Polyamines and *Fusobacterium nucleatum* in Periodontitis in the Context of Tregs and Th17 Cells

### Polyamine biology and immunomodulation

Polyamines are necessary for immune cell activation and proliferation, and they modulate proinflammatory cytokine production [14,18,19,57]. Critically, polyamines determine the functional fates of T helper subsets through a mechanism requiring eIF5A. eIF5A undergoes hypusination, wherein spermidine donates an aminobutyl group to a specific lysine residue, generating hypusine that is essential for translation of specific immune effector mRNAs. This positions the polyamine–eIF5A axis as a master regulator of T helper cell identity. Our laboratory has shown that the putrescine–eIF5A axis is a cardinal determinant of oral mucosal T helper dysregulation and chronic inflammation in people living with HIV (PLWH) [7,17-19,21,58]. Polyamines do not induce FOXP3^+^Tregs but render existing FOXP3^+^Tregs dysfunctional, increasing the frequency of FOXP3^+^PD-1^+^IFN-*γ*^+^TregDys cells in PLWH [7,21]. Polyamines enhance expression of eIF5A and hypusinated-eIF5A, a key indicator of polyamine dysregulation [7,11,13,15,21]. Consistent with our previous characterization of TregDys [55,56,59], polyamines also upregulate Amphiregulin (AREG) in Tregs and contribute to TregDys expansion as shown by increased KI-67 expression. Together, these data uncover the function of excessive polyamines in causing T helper infidelity, involving the induction of TregDys cells that retain FOXP3 but acquire a pro-inflammatory, non-suppressive phenotype. The loss of functional Tregs in a similar manner may be critical in periodontitis, as Treg dysfunction is known to remove the brake on Th17 differentiation and osteoclastogenesis, enabling expansion of pathogenic Th17 cells. These cells signal through IL-17A on stromal targets, producing IL-6, IL-8, TNF-*α*, CXCL1, and CXCL2, sustaining neutrophil recruitment, tissue destruction, and osteoclast activation [43,44,47]. In addition, polyamine catabolism, mediated by spermidine/ spermine N^1^-acetyltransferase (SSAT) and spermine oxidase (SMOX), generating reactive oxygen species (ROS) including H_2_O_2_ and toxic aldehydes such as acrolein as byproducts [60-62]. Polyamine biosynthesis also competes with nitric oxide (NO) synthesis for the common substrate arginine—a competition with profound implications for immune cell function. Polyamine biosynthesis competition with NO biosynthesis is implicated in macrophage polarization and the inflammatory microenvironment of the periodontal pocket. Whether T cell- dysregulation and polyamine-catabolism pathways contribute to immunopathology of periodontitis are unknown.

### Polyamines in the periodontal microenvironment

The earliest evidence linking polyamines to periodontitis was provided by Lamster *et al*. (1987), who demonstrated that GCF from untreated periodontitis sites contained putrescine at concentrations approximately 10^4^ times greater than serum levels. Putrescine was detected in all periodontitis samples and 12 of 15 gingivitis samples, with significant increases in periodontitis when compared to post-treatment and gingivitis [20]. Spermidine and spermine were detected only occasionally in GCF, suggesting putrescine plays a dominant pathological role. Stage-dependent increase in total polyamine levels, with concomitant elevation of ODC protein levels and correlation with reduced antioxidant capacity and increased acid phosphatase activity has been observed in periodontitis patients [20,63]. While elevated putrescine levels are linked to PD, which are substantially reduced following treatment [20,63], no study to date has delineated the origin of polyamines in the periodontal microenvironment, nor investigated its role in shaping innate or adaptive immune responses in PD.

### *Fusobacterium nucleatum* as a polyamine producer and immune modulator

*F. nucleatum* occupies a uniquely central role in oral biofilm ecology, functioning as a critical "bridging organism" that links early colonizers such as *Streptococcus* spp. to late-stage keystone pathogens including *Porphyromonas gingivalis*, thereby orchestrating the transition from a commensal to a dysbiotic, disease-driving microbiome [64]. *F. nucleatum* exhibits potent pro-inflammatory capacity through strong adhesion to epithelial cells and the extracellular matrix, and robust activation of innate immune signaling via TLR2/4 and NF-*κ*B pathways [9]. The FAD-I lipoprotein of *F. nucleatum* mediates human beta-defensin 2 induction through TLR-1/2 and TLR-2/6, illustrating sophisticated mechanisms of innate immune engagement [65]. Moreover, *F. nucleatum* acts as a predominant direct polyamine producer, making it an important immune modulator [7-9]. LC-MS analysis of *F. nucleatum* lysates and bacterial supernatants reveal higher concentrations of SPD and PUT in *F. nucleatum* cells cultured with ornithine [66]. Analysis of spent medium revealed substantial depletion of ornithine alongside increased putrescine concentration, confirming that *Fusobacterium* spp. effectively utilizes ornithine for growth and putrescine production. These findings support the possibility that *Fusobacterium* spp. abundance during dysbiosis contributes to elevated putrescine levels, which in turn may further support *Fusobacterium* colonization in a positive feedback loop perpetuating periodontitis. Beyond *F. nucleatum*, *Bacteroides* spp. are also implicated in the synthesis of putrescine and spermidine in the periodontal environment.

## Polyamine Dysregulation in IBD: Alterations in Metabolism and Function

Dysregulation of polyamine metabolism is also increasingly recognized as both a consequence of and contributor to chronic inflammation and mucosal injury in IBD [67-71]. Studies have shown that ODC activity and tissue levels of putrescine and spermidine are significantly elevated in the inflamed mucosa of IBD patients [72], reflecting increased epithelial turnover and immune activation in response to mucosal injury. However, chronic activation of polyamine synthesis leads to enhanced oxidative stress through catabolic enzymes SSAT and SMOX, which generate ROS and toxic aldehydes [15,73]. These oxidative byproducts exacerbate epithelial damage and perpetuate inflammation, underscoring the dual, protective yet potentially harmful nature of polyamines in intestinal homeostasis and inflammation [22,73,74]. At physiological levels, polyamines promote gut epithelial cell proliferation, migration, and tight junction assembly, facilitating mucosal healing and barrier maintenance [22,73,74]. Spermidine, in particular, enhances autophagy and mitochondrial function through eIF5A hypusination and mTOR inhibition, mechanisms that mitigate inflammation and foster epithelial restitution. Given that individual polyamines exert context-dependent effects on cellular function, absolute abundance alone may not adequately capture their biological significance. For instance, excessive putrescine accumulation combined with reduced spermidine has been implicated in immunopathological processes in certain contexts. Therefore, polyamine ratios, such as Putrescine/Spermidine and Putrescine/Spermine must be assessed in addition to individual polyamine concentrations, as these ratios may more accurately reflect the underlying biochemical and immunological milieu. Polyamine transporter mechanisms, instead of merely their levels should also be interpreted with caution. Mice lacking SLC7A2, a polyamine transporter, are more susceptible to DSS-induced colitis, with increased weight loss, higher mortality, and more severe tissue damage. Consistently, SLC7A2 expression is reduced in the colonic mucosa of patients with active UC or CD, which may contribute to mucosal injury [75,76]. Loss of SLC7A2 also leads to exaggerated chemokine production with a shift from a Th1 to Th17 response in DSS-treated mice [77]. Moreover, reduced SLC7A2 expression in a chronic IBD model contributes to the risk of developing colitis-associated colon cancer, positioning diminished SLC7A2 as a biomarker of IBD pathogenesis that implies impairment of arginine uptake and a potentially beneficial role for arginine supplementation. Conversely, excessive polyamine oxidation amplifies inflammatory cascades via hydrogen peroxide production and may skew macrophage polarization toward a proinflammatory M1 phenotype [78-80]. Thus, polyamine homeostasis, rather than absolute levels, determines whether their effects are reparative or pathological in immune microenvironment.

### *Fusobacterium nucleatum* in IBD: dysbiosis and intestinal inflammation

The role of *F. nucleatum* extends beyond the oral cavity to the gastrointestinal tract, where it has been implicated in intestinal inflammation and pathogenesis [81,82]. In DSS-induced colitis, administration of *F. nucleatum* significantly worsened disease severity [83]. *F. nucleatum* secretes outer membrane vesicles (OMVs) that promote intestinal inflammation, demonstrating its capacity to modulate immune responses at sites distant from primary infection [81]. This OMV-mediated pro-inflammatory activity activates NF-*κ*B and STAT-3 pathways in intestinal epithelial cells, inducing cytokine cascades that parallel those observed in the periodontal pocket [68,81,84]. In the context of IBD specifically, *F. nucleatum* facilitates ulcerative colitis through activation of IL-17F signaling to NF--B via upregulation of CARD3 expression [84]. It also aggravates the progression of colitis by regulating M1 macrophage polarization via the AKT2 pathway [83]. The detection of *F. nucleatum* in IBD-associated dysbiotic communities suggests that its polyamine-producing capacity and pro-inflammatory properties contribute to intestinal immunopathology through mechanisms analogous to those operating in periodontitis. *F. nucleatum*'s ability to produce putrescine via the ornithine decarboxylase pathway, validated in our laboratory through anaerobic culture experiments and targeted LC-MS analysis [7,8,21], positions it as a potential contributor to the elevated luminal polyamine pools observed in IBD patients. The resulting polyamine-rich intestinal microenvironment could drive TregDys induction and T cell expansion through the same eIF5A hypusination mechanism demonstrated in the oral mucosa [21], perpetuating the chronic mucosal inflammation characteristic of IBD, but these mechanisms remain unstudied in the context of IBD.

## Microbiota–polyamine-T cell Interactions in IBD

In addition to epithelial cell-derived polyamine pools, the gut microbiota is a major contributor to luminal polyamine concentrations [15]. Germ-free mice have markedly lower intestinal polyamine concentrations than conventionally housed mice, demonstrating that the microbiota is a primary source of luminal polyamines [85-87]. Commensal bacteria such as *Bacteroides* and *Bifidobacterium adolescentis* produce polyamines that regulate epithelial proliferation and immune tolerance [22,88-90]. Dysbiosis in IBD alters microbial polyamine synthesis, leading to aberrant mucosal immune responses and delayed tissue repair. Notably, microbial genes for polyamine biosynthesis are upregulated in IBD patients, suggesting a compensatory or pathogenic adaptation to inflammation [72,91,92]. This parallels the observation in periodontitis and PLWH that salivary putrescine levels strongly correlate with *Fusobacterium* spp. enrichment, implicating dysbiotic microbial communities as primary drivers of polyamine dysregulation in oral mucosal surfaces [21]. The mechanistic cascade in IBD partly mirrors that proposed for periodontitis: dysbiosis enriches the gut microbiome with polyamine-producing organisms including *Fusobacterium* and *Bacteroides* spp.; elevated luminal putrescine and other polyamines alter gut immune homeostasis [93]. Under physiological conditions, spermidine is known to be immunomodulatory, inducing Treg differentiation to maintain gut immune tolerance [94], and restoring mucosal immune homeostasis via mTOR-dependent autophagy and eIF5A hypusination [95-98]. However, the consequences of excessive polyamines in the context of dysbiosis, their impact on mucosal immune cells and CD4^+^T cells, the destabilization of Tregs into TregDys cells that lose suppressive function, and the resulting expansion of pathogenic T cells driving mucosal inflammation and epithelial barrier disruption remain to be fully elucidated [15]. These represent important directions for future investigation.

## Conclusion

**Figure 1.**
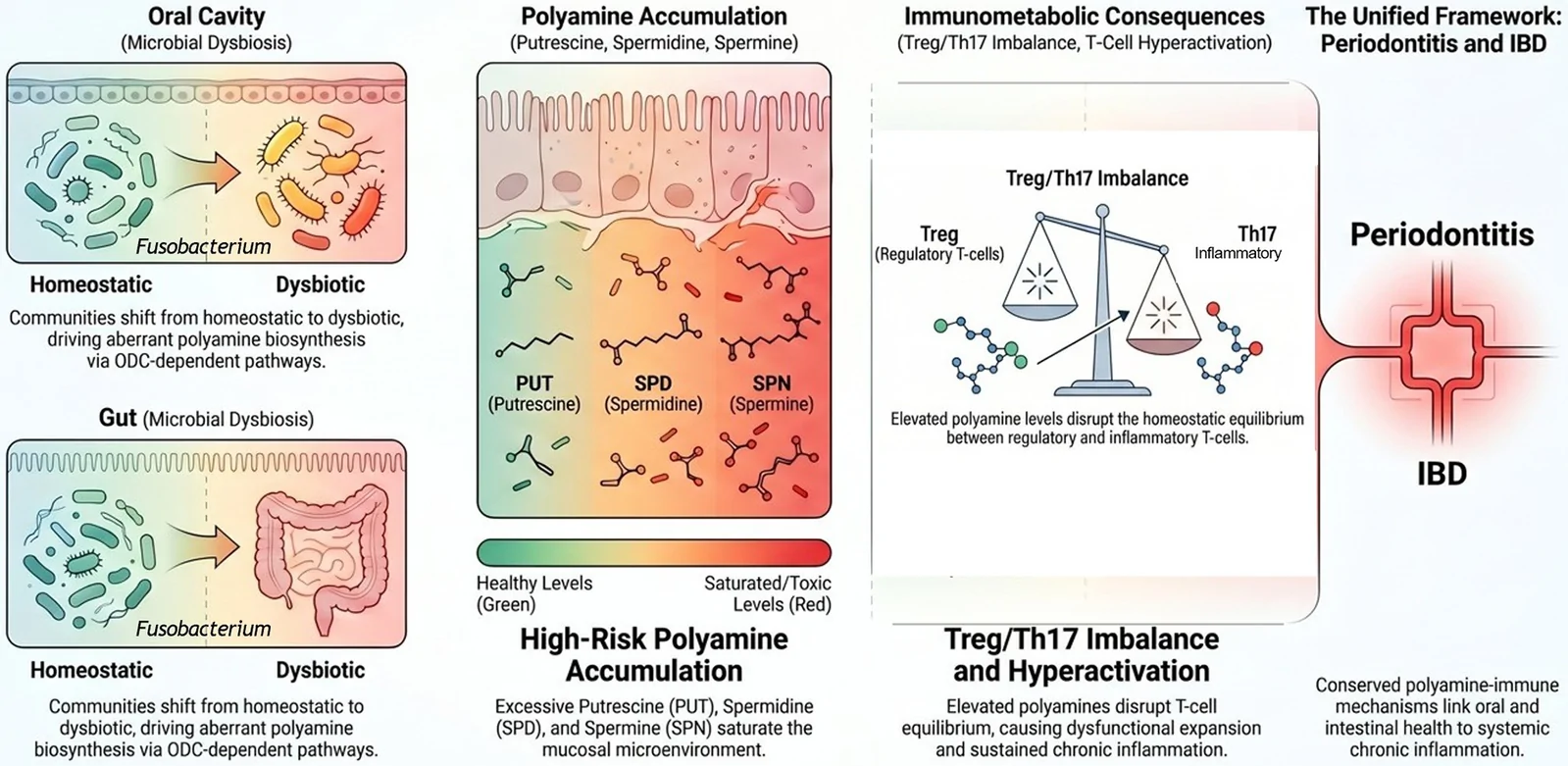
The polyamine-mediated oral-gut inflammatory axis.

The central thesis of this review is that dysbiotic microbial communities, particularly those enriched with *Fusobacterium nucleatum*, drive a cascade of immunometabolic dysregulation that converts protective immune responses into destructive immunopathology (Figure 1). The shared mechanistic nodes— dysbiosis-driven polyamine excess, eIF5A hypusination, and disruption of the Treg/Th17 balance—suggest that therapeutic strategies developed in IBD may be applicable to periodontitis, and vice versa. Precision modulation of polyamine metabolism, rather than broad suppression, emerges as the key therapeutic principle: harnessing the anti-inflammatory and autophagy-promoting properties of spermidine while preventing the oxidative stress and TregDys induction associated with putrescine excess. Context-specific immunomodulation that preserves protective Th17 responses while selectively suppressing pathogenic T cells and restoring functional Tregs is preferable to global cytokine inhibition.

Future research should prioritize: (1) delineating the precise molecular mechanisms by which polyamines modulate eIF5A hypusination and mTOR signaling in mucosal Tregs and Th17 cells; (2) validating polyamine-based biomarker panels and ratios in large-scale clinical cohorts; and (3) exploring the therapeutic potential of ODC inhibitors and spermidine supplementation across mucosal inflammatory diseases. Collectively, the evidence reviewed here supports a unified oral-gut axis model in which dysbiosis, polyamine dysregulation, and Treg/Th17 imbalance form a self-reinforcing pathogenic triad amenable to targeted immunometabolic intervention.
